# Influence of concentration, irrigation method, and root canal third on intratubular penetration of sodium hypochlorite – a broad statistical analysis 

**DOI:** 10.4317/jced.60955

**Published:** 2023-11-01

**Authors:** Ricardo Machado, Breno Nantes, Diego-Augusto Guimarães, Stella-Maria-Glaci Reinke, Eduardo-Donato-Eing-Elgelke Back, Daniel Comparin, Sérgio-Aparecido Ignácio, Luiz-Pascoal Vansan

**Affiliations:** 1 DDS, MSc, PhD. Clinical practice limited to Endodontics, Navegantes, Santa Catarina, Brazil; 2 DDS, MSc. Department of Restorative Dentistry (Endodontics), School of Dentistry of Ribeirão Preto, University of São Paulo – FORP/USP, Ribeirão Preto, São Paulo, Brazil; 3 DDS, MSc, PhD. Clinical practice limited to Endodontics, Blumenau, Santa Catarina, Brazil; 4 DDS, MSc. Clinical practice limited to Endodontics, Joinville, Santa Catarina, Brazil; 5 DDS, MSc. Clinical practice limited to Endodontics, Francisco Beltrão, Paraná, Brazil; 6 DDS, MSc, PhD. Department of Biostatistics, School of Life Sciences, Faculty of Dentistry, Pontifical Catholic University of Paraná – PUC/PR, Curitiba, Paraná, Brazil

## Abstract

**Background:**

The permanence of microorganisms in the root canal system represents the main cause of endodontic failure. Considering the impossibility of effective action of the endodontic files in ramifications of the main canal and mainly inside the dentinal tubules, a better understanding of the irrigation dynamics to enhance endodontic prognosis is essential. Objective: To evaluate the depth of intratubular penetration values of sodium hypochlorite (NaOCl) (dependent variable) by comparing different concentrations, methods of irrigation, and root canal thirds (independent variables) and to investigate the existence of interactions among them, capable of influencing the dependent variable.

**Material and Methods:**

40 roots from extracted human maxillary central incisors were stained and instrumented according to four irrigation protocols (n. 10): conventional irrigation (CI) at each use or change of instrument, and final irrigation with 5ml of 2.5% or 5.25% NaOCl, with or without passive ultrasonic irrigation (PUI), respectivelly. Measurements based on stereomicroscopic images were obtained, and the data were subjected to statistical analysis (*p*< 0.05).

**Results:**

The highest depth values of intratubular penetration of NaOCl were observed in the cervical third, at 5.25%, and by PUI. When only two independent variables were analyzed in association, the highest penetration depth values of NaOCl were obtained at 5.25%, regardless of irrigation method, at 5,25%, in the cervical third, and; in the cervical third, despite of irrigation method. Considering the three independent variables simultaneously, the highest depth values of intratubular penetration of NaOCl were observed in the cervical third, at 5.25%, no matter the irrigation method. The interaction between the independent variables on the penetration depth values of NaOCl was only confirmed considering the irrigation method and root canal third.

**Conclusions:**

Intratubular penetration of NaOCl was influenced by the three independent variables individually and when the irrigation method and root canal third were considered simultaneously.

** Key words:**Dentinal tubules, Depth, Disinfection, Irrigation, Root canal system, Sodium hypochlorite.

## Introduction

The main goal of Endodontics is to keep natural teeth by preserving or restoring the health of periapical tissues. In necrotic teeth, the pulp cells are compromised, thus favoring root canal system (RCS) infection (1). Even if no periapical lesion is visualized radiographically, it may be present (2); therefore, treatment is performed to restore the health of the local tissues (1).

The biomechanical preparation is the main factor responsible for endodontic infection control (3). It is performed by associating the use of instruments and auxiliary chemical substances, generally introduced into the root canal through a needle and a syringe – conventional irrigation (CI) (4). Although mechanical and chemical cleaning occurs inseparably, enlargement and shaping of the root canal are known to be achieved by using endodontic files; meanwhile, chemical cleaning is performed by active substances in contact with the root canal walls. Both actions (mechanical and chemical cleaning) are complemented by the physical cleaning mechanism that happens during the flow and reflux of the irrigant (4,5).

Antimicrobial strategies during endodontic interventions are much more effective in the main root canal. However, the maintenance of remnants of microorganisms, including in the dentinal tubules, has previously been evidenced by several studies (6,7), being recognized as an important factor responsible for endodontic failure (8). According to Ando and Hoshino in 1990 (9), the depth of intratubular penetration of microorganisms can vary from 150µm to half the distance between the root canal walls and the cementum-dentin junction. Considering that after 20 minutes of exposure, the highest depth value of intratubular penetration of a 6% sodium hypochlorite (NaOCl) solution heated to a temperature of 45ºC is around 300µm (10), the search for more effective strategies for disinfecting dentinal tubules in greater depth has been encouraged (11).

Passive ultrasonic irrigation (PUI) is one of the most studied methods to optimize the disinfection of the RCS. The energy released by inserts during PUI produces cavitation and acoustic streaming, resulting in microbubbles and hydrodynamic waves that promote agitation of the liquid against the root canal walls, favoring their cleaning and the penetration of the irrigating solution into the dentinal tubules (12).

Pécora, *et al*. (13) described a histochemical technique for the analysis of dentin permeability based on a methodology initially proposed by Feigl in 1958 (14). Copper represents the cation indicating the extent of depth of dentin permeability in a 10% copper sulfate solution. The complexation of rubeanic acid in a 1% rubeanic acid alcohol solution reveals the presence of copper ions, resulting in dentin pigmentation, which varies from dark blue to black (copper rubeanate). The method’s main advantages include its easy reproducibility, the fact that copper ions have molecules smaller than dyes, and the absence of potential systemic damage caused by radioisotopes (13).

Extensive research using several analysis methodologies has previously been conducted to compare the depth of intratubular penetration of different endodontic irrigants at different concentrations, temperatures, chemical formulation, and distinct activation methods in the three root canal thirds (10,15-17). Nevertheless, no study has been conducted to analyze these variables in isolation, paired (two by two), and associated with one another, in addition to investigating probable interactions that influence the penetration depth of NaOCl through a broad statistical approach.

The aims of the present study were: 1) to evaluate the depth of intratubular penetration values of NaOCl (dependent variable), comparing two concentrations (2.5 and 5.25%) and two irrigation methods (CI and PUI) in the three root canal thirds (cervical, middle and apical) (independent variables), and; 2) to investigate whether there were interactions between these variables, capable of influencing the depth of penetration of the irrigant, by using a comprehensive statistical analysis performed with data obtained by the histochemical method proposed by Pécora, *et al*. (13). Bearing this in mind, the following null hypotheses were considered: 1) the depth of intratubular penetration of NaOCl would not be altered by concentration, irrigation method, and root canal third, and 2) there would be no interactions between these variables capable of influencing the depth of intratubular penetration of the irrigant.

## Material and Methods

-Approval by the Research Ethics Committee, selection, and preparation of specimens

For the development of this study, after authorization issued by the Research Ethics Committee of the Ribeirão Preto School of Dentistry – FORP/USP (CAAE: 14962013.4.0000.5419), 40 extracted human maxillary central incisors were selected. The teeth were supplied by the Tooth Bank of the same Institution and had a single canal, complete rhizogenesis, without abrupt curvatures, resorption processes, or previous endodontic treatments. These characteristics were confirmed visually and on radiographs taken in the mesiodistal and buccolingual directions. The specimens selected were stored in 0.1% thymol solution at 48°C and washed in running water for 24 hours before use (18). After this, the roots were cleaned with a P20 ultrasonic insert (Helse, Santa Rosa de Viterbo, São Paulo, Brazil) coupled to a Profi II Ceramic equipment (Dabi Atlante Ltda, Ribeirão Preto, São Paulo, Brazil) and the coronal portions were removed using a double-sided disc (KG Sorensen, Barueri, São Paulo, Brazil) to obtain root segments approximately 16 mm long.

After the canal was irrigated with 5 ml of distilled and deionized water using a Navitip needle and a 5 ml plastic syringe (Ultradent, South Jordan, Utah, United States), it was initially explored through an n. 20 K-FlexoFile (Dentsply-Maillefer, Ballaigues Switzerland). Then, the specimens were placed in a vacuum desiccator (Laborglass, São Paulo, São Paulo, Brazil) for 120 minutes to allow complete dehydration. After this, they were immersed in a copper sulfate solution and placed in a desiccator coupled to a vacuum pump (Quimis, Diadema, São Paulo, Brazil / 27Kg/cm2) for 15 minutes to remove air from the main canal to enable more extensive penetration of the solution mentioned above. Subsequently, the specimens remained immersed in this solution for another 75 minutes at ambient atmospheric pressure, and the root surface and the canal were then dried with paper towels and n. 20 absorbent paper cones (Dentsply-Maillefer), in that order. Finally, all specimens were immersed in a rubeanic acid solution, transferred to and stored in the desiccator coupled to a vacuum pump, and exposed to ambient atmospheric pressure for 15 and 225 minutes, respectively.

-Biomechanical preparation 

Before performing biomechanical preparation, each root was wrapped with a thin layer of hydrophilic vinyl imprint vinyl polysiloxane (Oranwash; Zhermack SpA, Rovigo, Italy) to simulate the periodontal ligament space and embedded in acrylic resin blocks (Techno Tray-P, Protechno, Girona, Spain) (19) to avoid the extravasation of the irrigation solution beyond the apical foramen. In each specimen, the root canal orifice was prepared with Largo n. 2 (Dentsply-Maillefer) and n. 3082 (KG Sorensen), and the cervical and middle thirds were prepared with Gates-Glidden n. 4, 3, and 2 burs (Dentsply-Maillefer) according to the principles of the Crown-Down technique. After this, the working length (WL) was established by introducing an n. 15 K-FlexoFile (Dentsply-Maillefer) until the apical foramen, subtracting 1 mm from this measurement. The anatomical diameter was determined by inserting K-FlexoFile instruments of the first series (Dentsply-Maillefer) in ascending order, up to the first instrument that fitted to the walls of the root canal in the WL (20). The biomechanical preparation was conducted following the principles of the Crown-Down technique using sequential files with respective diameters immediately greater than the anatomical instrument. During this phase, the specimens were randomly divided into four groups (n = 10) according to the irrigation protocol:

• CI with 5ml of 2.5% NaOCl at each use or change of instrument, and final irrigation with 5ml of 2.5% NaOCl for 30 seconds*;

• CI with 5ml of 2.5% NaOCl at each use or change of instrument*, and PUI** with 5ml of 2.5% NaOCl for 30 seconds*;

• CI with 5ml of 5.25% NaOCl at each use or change of instrument, and final irrigation with 5ml of 5.25% NaOCl for 30 seconds*;

• CI with 5ml of 5.25% NaOCl at each use or change of instrument*, and PUI** with 5ml of 5.25% NaOCl;

*Procedure performed with a Navitip irrigation tip (Ultradent) inserted at -2mm from the WL.

**Procedure performed with an Irrisonic ultrasonic insert (Helse), inserted at -2mm from the WL, at a power of 20kHz.

Subsequently, the root canal was dried with absorbent paper cones (Dentsply-Maillefer) with a diameter corresponding to the final instrument used during the biomechanical preparation.

-Analysis of intratubular penetration values of NaOCl

Each specimen was sectioned horizontally at 2, 8, and 14mm from the apex using a low-speed steel cutting disc (Isomet-Buehler, Lake Bluff, IL, USA). Three slices per root were obtained, resulting in 120 slices. A standard polishing procedure using SiC paper (200,300,400,600) followed by three μm diamond paste was employed on the cervical surface of each slice to produce a high-reflection surface, and each slice was observed in a high-resolution stereomicroscope to acquire images at 1048x1048 pixels, covering the entire root surface. For each image, the areas corresponding to discolored dentin and the perimeter of the root canal were measured using the AxioVision Software 4.11 (Carl Zeiss, Jena, Germany). By subtracting the area corresponding to the perimeter of the root canal from the region corresponding to the discolored dentin, intratubular depth penetration of the irrigant was determined in mm2.

-Statistical analysis

Considering the objectives of the present study, the assumption of normality of the data was initially evaluated using the Shapiro-Wilk test, which indicated that all data had a normal distribution (*p* > 0.05). Therefore, the 3-way parametric analysis of variance (ANOVA) test (full factorial model) was applied to identify statistical differences concerning the mean values of the dependent variable studied (intratubular penetration depth of NaOCl), according to independent variables: concentration (2.5% and 5.25%); irrigation method (CI and PUI) and root canal third (cervical, middle and apical), as well as to analyze the paired and associated interaction between them. Since there were statistically significant differences, the Tukey HSD and Games-Howell parametric tests for multiple comparisons were applied for homogeneous and heterogeneous variances. The Levene test was used to identify the homogeneity of variances in the depth of intratubular penetration values of NaOCl among the irrigation protocols. The significance level was set at 0.05, and all analyses were performed using the IBM SPSS Statistic 25.0 software (IBM, Armonk, NY, USA) (21).

## Results

-Data availability

The complete statistical analysis of this research can be ac-cessed at: https://1drv.ms/f/s!AnuvHAhxDtNGg75jJv8e-jlJeQcGcqg?e=qNqX9X.

-Depth of intratubular penetration values of NaOCl, considering the irrigation method, concentration, and root canal third, individually

**Figure 1:**
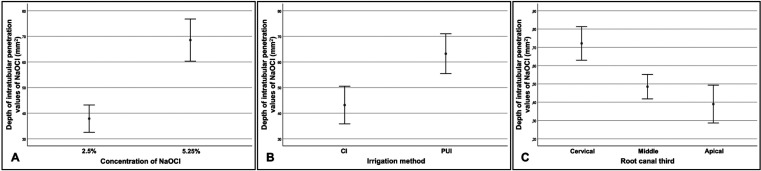
Confidence intervals (95%) of the values referring to the mean depth of intratubular penetration of NaOCl, considering the concentration (A), irrigation method (B), and root canal third (C), separately.

Given the normal distribution of the data, comparisons among the mean values of intratubular penetration depth of NaOCl, considering the concentration, irrigation method, and root canal third individually, were performed using the 3-way parametric analysis of variance (ANOVA) test (full factorial model). This test showed F values of 68.88, 29.41, and 28.46, respectively, and *p* < 0.01, with a test power of 99.99% determined based on the sample size. Thus, the existence of statistically significant differences was proved by comparing concentrations of the irrigant (2.5% and 5.25%), irrigation methods (CI and PUI), and root canal thirds evaluated (cervical, middle, and apical). The highest depth values of intratubular penetration of NaOCl were observed in the cervical root canal thirds (0.72 ± 0.28 mm2), using the product at the concentration of 5.25% (0.68 ± 0.31 mm2) and in an activated manner (PUI) (0.63 ± 0.30mm2) (*p* < 0.01). The lowest values occurred in the apical root canal thirds (0.38 ± 0.32mm2), with no statistically significant differences in comparison to the middle root canal thirds (0.48 ± 0.20mm2) (*p* ˃ 0.05) (Table 1, Fig. 1A-C).

**Table 1:**
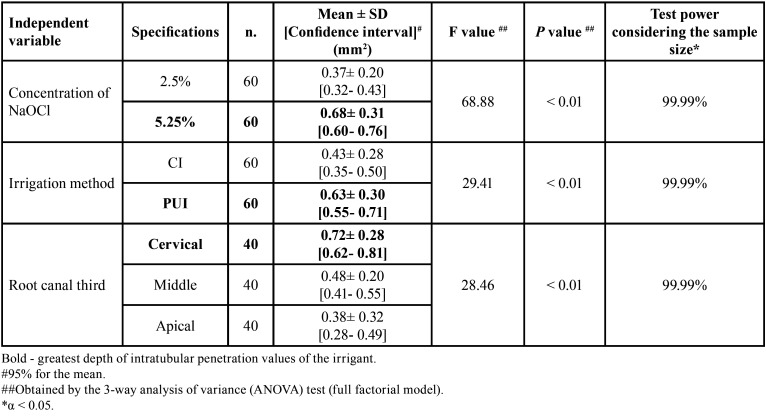
Statistical data regarding the depth of intratubular penetration values of NaOCl, considering independent variables individually (concentration, irrigation method, and root canal third).

-Depth of intratubular penetration values of NaOCl, considering concentration/irrigation method, concentration/root canal third, and irrigation method/root canal third

**Figure 2:**
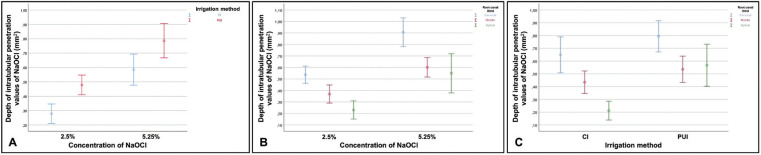
Interval of confidence (95%) of the mean depth of intratubular penetration of NaOCl, considering concentration/irrigation method (A), concentration/root canal third (B), and irrigation method/root canal third (C).

As regards the differences between the mean values of intratubular penetration depth values of NaOCl considering concentration/irrigation method, concentration/root canal third, and irrigation method/root canal third, the 3-way analysis of variance (ANOVA) test (full factorial model), showed F values of 21.33, 19.71 and 12.01, respectively, and *p* < 0.01, with a test power of 99.99% determined based on the sample size. The highest penetration depth values of NaOCl were obtained by the following combinations, without significant statistical differences between them (*p* ˃ 0.05): 5.25%/PUI (0.78 ± 0.31mm2) and 5.25%/CI (0.58 ± 0.28mm2); 5.25%/cervical root canal third (0.90 ± 0.26mm2), and; CI/cervical root canal third (0.64 ± 0.30mm2) and PUI/cervical root canal third (0.79 ± 0.26mm2) (Table 2, Fig. 2A-C). All comparisons are shown in Table 3- Table 3 cont.-1.

**Table 2:**
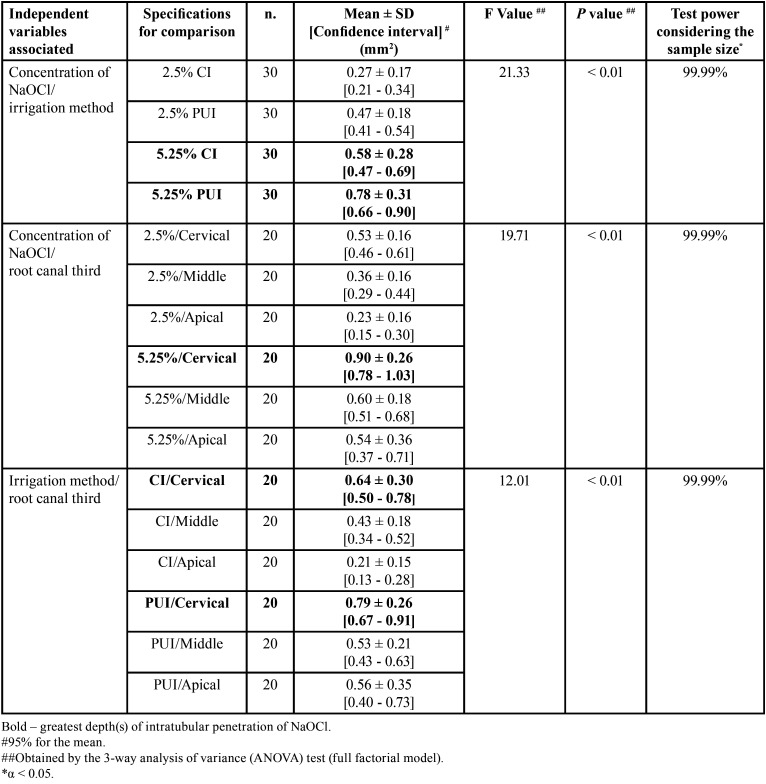
Statistical data regarding the depth of intratubular penetration of NaOCl, considering concentration/irrigation method, concentration/root canal third, and irrigation method/root canal third.

**Table 3:**
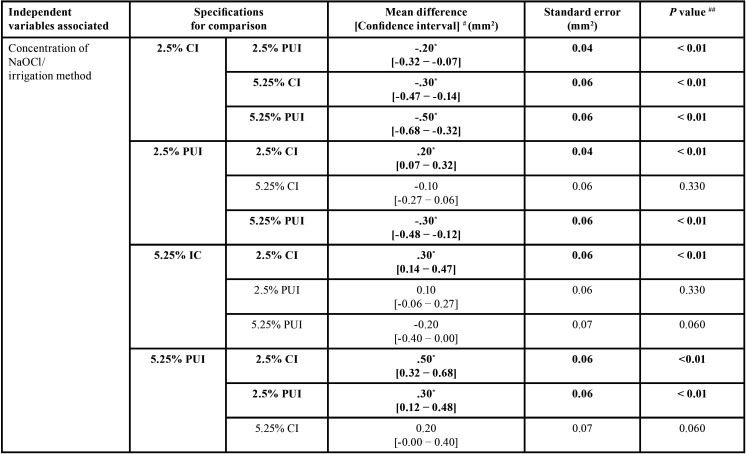
Statistical data regarding the depth of intratubular penetration of NaOCl, considering concentration/irrigation method, concentration/root canal third, and irrigation method/root canal third (all comparisons).

**Table 3 cont.**
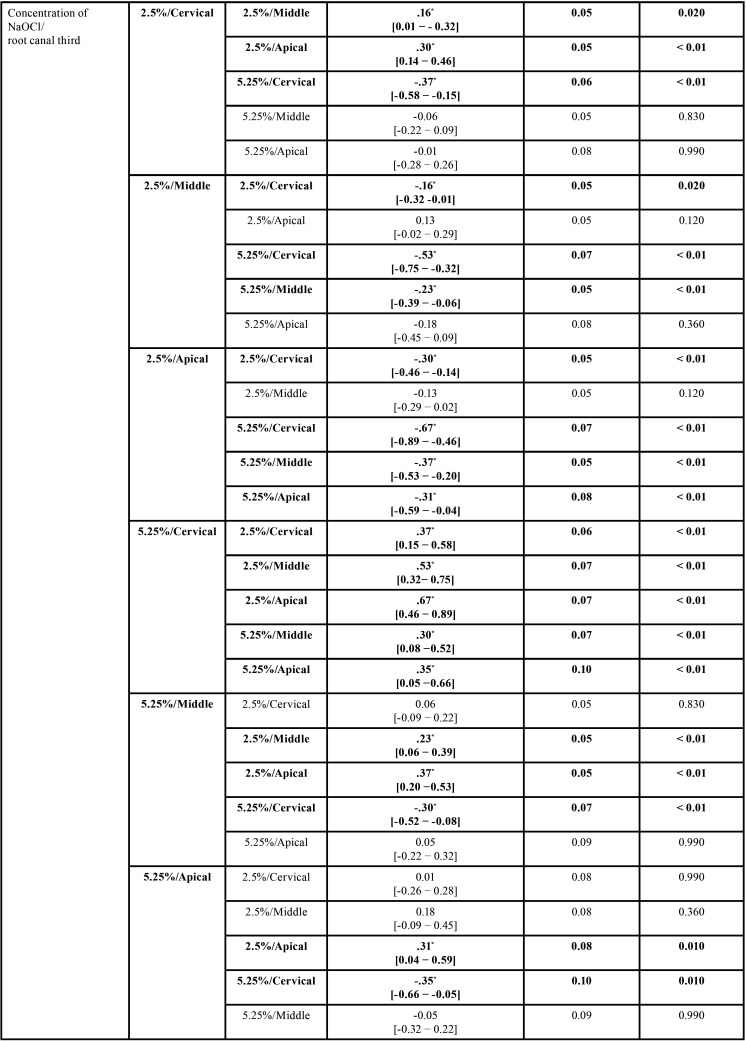
Statistical data regarding the depth of intratubular penetration of NaOCl, considering concentration/irrigation method, concentration/root canal third, and irrigation method/root canal third (all comparisons).

**Table 3 cont.-1:**
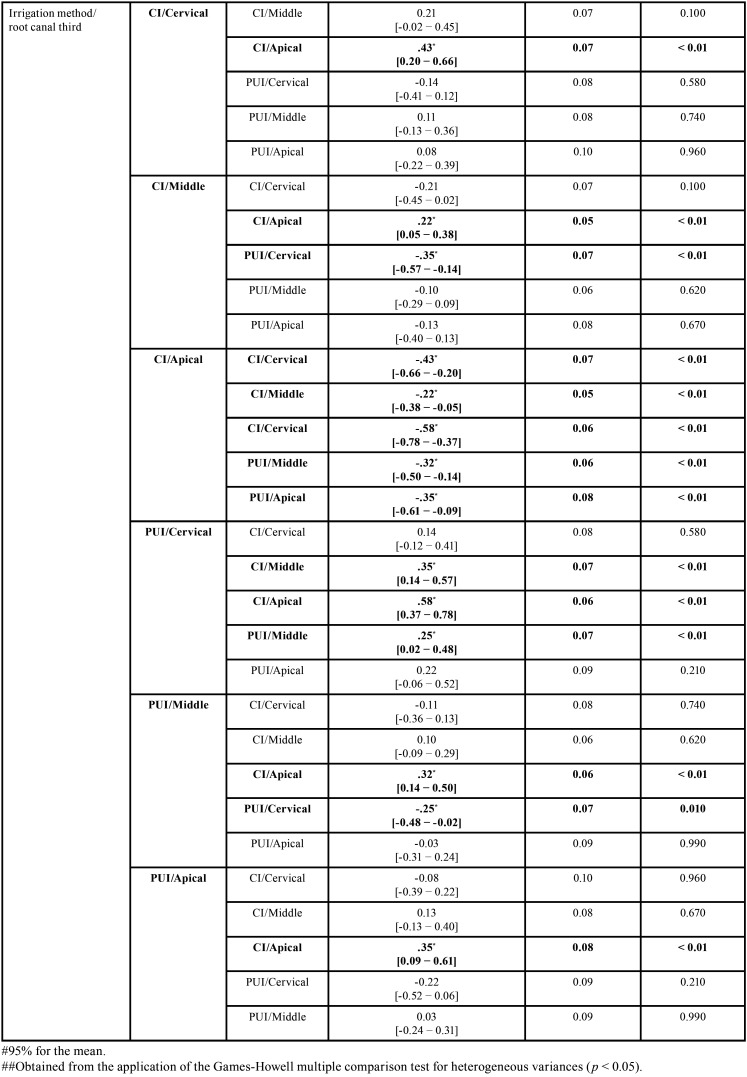
Statistical data regarding the depth of intratubular penetration of NaOCl, considering concentration/irrigation method, concentration/root canal third, and irrigation method/root canal third (all comparisons).

-Depth of intratubular penetration of NaOCl considering the irrigation method, concentration, and root canal third, simultaneously

**Figure 3:**
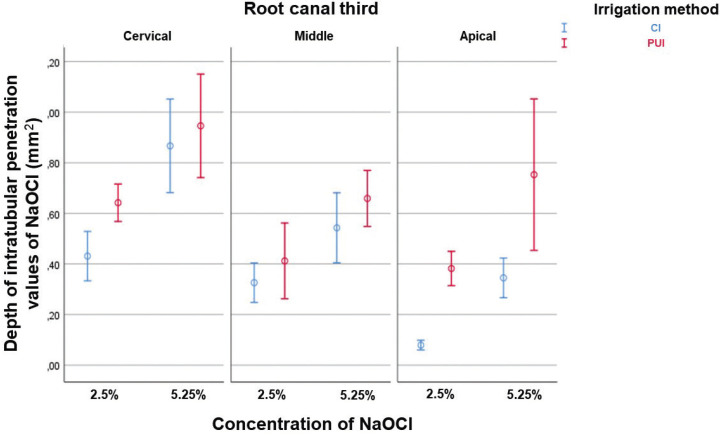
Depth of intratubular penetration of NaOCl, considering the irrigation method, concentration, and root canal third, simultaneously.

About the differences between the mean values of intratubular penetration depth of NaOCl, considering the three independent variables simultaneously, the F value was 15.31 and *p* < 0.01, with test power considering the sample size of 99.99%, indicating that there were statistically significant differences. The highest depth values of intratubular penetration of the irrigant were observed in the cervical root canal thirds, using it at the concentration of 5.25%, without statistically significant differences between irrigation methods (PUI/5.25%: 0.94 ± 0.28mm2; CI/5.25%: 0.86 ± 0.25mm2 (*p* > .05); PUI/2.5%: 0.64 ± 0.10mm2; CI/2.5%: 0.43± 0.13mm2). (*p* < 0.05). In the middle root canal third, there was no statistically significant di-fference when irrigation methods and concentrations of irrigant were compared (PUI/5.25%: 0.65 ± 0.15mm2; CI/5.25%: 0.54 ± 0.19mm2; PUI/2.5%: 0.41 ± 0.20mm2; CI: 2.5% 0.32± 0.10mm2) (*p* < 0.05). In the apical root canal third, the highest penetration depth values of NaO-Cl were obtained when it was used in higher concentration (5.25%), regardless of the irrigation method (PUI or CI) (*p* < 0.01) (Table 4, Fig. 3).

**Table 4:**
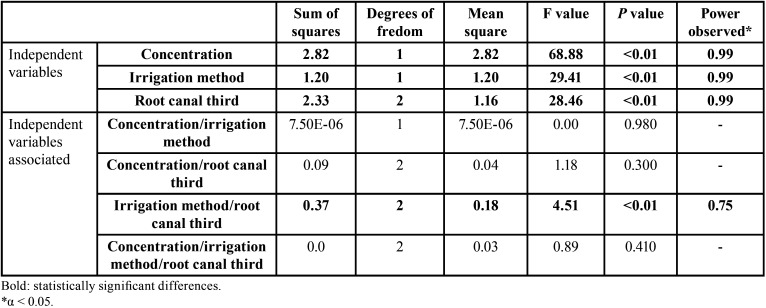
Statistical data regarding analysis of the interaction between independent variables (concentration. irrigation method, and root canal third) on the depth of intratubular penetration of NaOCl.

-Interaction between the irrigation method, concentration, and root canal third on the intratubular penetration depth of NaOCl

**Figure 4:**
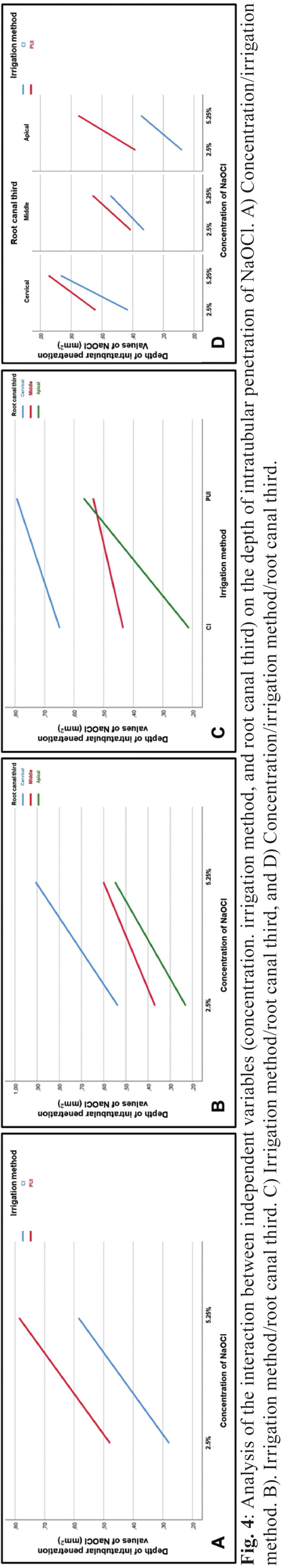
Analysis of the interaction between independent variables (concentration. irrigation method, and root canal third) on the depth of intratubular penetration of NaOCl. A) Concentration/irrigation method. B). Irrigation method/root canal third. C) Irrigation method/root canal third, and D) Concentration/irrigation method/root canal third.

Analysis of the interaction between the concentration of NaOCl and irrigation method, and the concentration of NaOCl and root canal third on the intratubular penetration depth of the irrigant, showed F values of 0.00 and 1.18, respectively, indicating their absence (*p* > 0.05) (Table 4, Fig. 4A,B, in this order).

When evaluating the interaction between the irrigation method and root canal third on penetration depth values of the irrigant, the F value was 4.51, with a test power of 75% determined based on the sample size, indicating interaction (*p* < 0.01) (Table 4, Fig. 4C).

Finally, when investigating the interaction between all independent variables simultaneously on the depth of intratubular penetration values of NaOCl, the value of F was 0.89, indicating no interaction (*p* > 0.05) (Table 4, Fig. 4D).

## Discussion

In most cases, endodontic failure is caused by remaining microorganisms inside the RCS (including the dentinal tubules), which can even reach up to half the distance between the root canal walls and the cementum-dentin junction (9). Considering that excessive intraradicular wear compromises the longevity of the endodontically treated teeth, eliminating or decreasing the number of microorganisms located deep inside the dentinal tubules is only feasible by promoting their direct contact with chemical substances (irrigation) (4), intracanal medication (22) and filling materials (23), or indirectly from the dentin alkalinization provided by the use of calcium hydroxide as intracanal dressing (22). Both antimicrobial processes are complemented by the nutritional scarcity obtained after conducting the root canal filling and definitive restoration (23).

Several methodologies have been used to assess the depth of intratubular penetration of NaOCl (17,24,25). In some studies, dyes have been added to the irrigant to enable further analysis by capturing confocal laser microscopy images (25). In other research, fluorescent solutions were used to stain the dentin, followed by measurements carried out through operative (24) and optical (17) microscopy images. These methodologies were not used in the present study since the oxidation reactions of NaOCl can affect the fluorescence of dyes and because the alternative solutions have significantly different properties (17). Therefore, we chose to use the stained dentin model proposed by Pécora, *et al*. (13). To achieve even more reliable results, strategies were used to mimic the clinical reality, such as using humans instead of bovine teeth (26) and traditional irrigation procedures instead of dentin blocks immersed in receptacles containing NaOCl (10). Furthermore, the quantity, frequency, and time of exposure of specimens to the auxiliary chemical substance were established based on routine clinical protocols (15). However, the sample selection method was the primary deficiency of this scientific investigation. Lack of knowledge about the age of the patients at the time of extractions, and the unknown volume and diameter of the dentinal tubules of the teeth used, prevented standardization of the baseline conditions of root dentin. The small sample size could also raise questions due to the greater possibility of Type 2 statistical errors (false negatives). However, the highly significant differences observed in most comparisons represent unquestionable proof of the results’ credibility. Nevertheless, the recommendation is to be cautious about transposing these findings to clinical reality since they were obtained in a “laboratory scenario” (17).

The isolated analyses of independent variables of this research showed that the highest depth values of intratubular penetration of NaOCl were observed in the cervical root canal thirds and when the concentration of 5.25% was actively used (PUI); therefore, the first null hypothesis established was rejected. Virdee, *et al*. (17) reported similar findings based on increases in NaOCl concentration (from 2% to 5.25%) and time of use (from 10 to 20 minutes). Although there were significant methodological differences, similar results were obtained by Zou, *et al*. (10), Palazzi, *et al*. (27), and Faria, *et al*. (26) which were justified by the higher level of chlorine ions released due to the use of more concentrated halogenated compounds, in addition to the constant renewal of the irrigant and more extended periods of using it (28). In the study of Virdee, *et al*. (17) these effects promoted higher depth values of intratubular penetration of the irrigant when using CI and manual dynamic activation (MDA) compared with PUI and sonic irrigation (SI). While the first two irrigation methods were being performed, a longer time may have been necessary to reach a higher depth value of intratubular penetration of the irrigant, which could have been “compensated” by increasing its concentration. Nevertheless, as the periods of use of the irrigant in the present study were the same during instrumentation and final irrigation, 2.5% NaOCl did not achieve results equivalent to those of 5.25% NaOCl, even when both concentrations were used by PUI and CI methods, respectively.

According to Muñoz & Cuadra, in 2012 (29), the irrigant is passively inserted into the root canal during CI. It immediately returns to the pulp chamber, making it difficult to achieve a significant depth of intratubular penetration. Furthermore, the shear forces and hydrodynamic pressures generated within the irrigant through PUI are significantly higher and more evenly distributed over more expansive areas of the root canal, promoting more intense contact with the root canal walls, thus potentiating its depth of intratubular penetration.(17) These arguments justify the lower penetration depth values of the irrigant through CI.

As occurred in the studies by Giardino, *et al*. (16) and Virdee, *et al*. (17), our results also showed higher depth values of intratubular penetration of the irrigant in the cervical root canal thirds. The argument most frequently found in the literature to justify this finding is the higher volume and larger diameter of the dentinal tubules present in the initial millimeters of the main root canal (30).

Considering concentration/irrigation method, concentration/root canal third, and irrigation method/root canal third, the highest penetration depth values of NaOCl were obtained by the following combinations, without statistically significant differences between them: 5.25%/PUI (0.78 ± 0.31mm2) and 5.25%/CI (0.58 ± 0.28mm2); 5.25%/cervical root canal third (0.90 ± 0.26mm2), and; CI/cervical root canal third (0.64 ± 0.30mm2) and PUI/cervical root canal third (0.79 ± 0.26mm2). When assessing the impact of the three independent variables together on the depth of intratubular penetration of the irrigant, the highest values were observed in the cervical root canal third of the specimens irrigated with 5.25% NaOCl, with no relevant statistical differences between PUI (0.94 ± 0.28mm2) and CI (0.86 ± 0.25mm2). Because of the higher volume and larger diameter of the dentinal tubules present in the cervical root canal third, the use of 5.25% NaOCl only, with the consequent release of a larger quantity of chlorine ions (28), appeared to have been sufficient to potentiate the depth of intratubular penetration, considering the use of the same period of exposure and the absence of statistical differences between the irrigation methods. In the middle root canal third, there was no statistically significant difference when the irrigation methods and concentrations of irrigant were compared (PUI/5.25%: 0.65 ± 0.15mm2; CI/5.25%: 0.54 ± 0.19mm2; PUI/2.5%: 0.41 ± 0.20mm2; CI: 2.5% 0.32 ± 0.10mm2); that is; both were irrelevant concerning the depth of intratubular penetration of irrigant in intermediate millimeters of the root canal. In the apical root canal third, the highest penetration depth values of NaO-Cl were obtained when it was used in higher concentration (5.25%), regardless of the irrigation method (PUI or CI) (*p* < 0.01). However, the trend towards achieving higher penetration depth values of NaOCl rose significantly because of its activation (PUI/5.25%: 0.75 ± 0.41mm2; CI/5.25%: 0.34 ± 0.10mm2; PUI/2.5%: 0.38 ± 0.09mm2; CI/2.5%: 0.07 ± 0.02mm2). Furthermore, the proven interaction between the irrigation method and root canal third demonstrated that PUI promoted relevant increases in the depth values of intratubular penetration of the irrigant in all root canal thirds, which were considerably higher in the apical third (0.35 mm2); therefore, leading to rejection of the second null hypothesis established.

Carmen Llena, *et al*. (31) evaluated the maximum depth and the percentage of intratubular penetration of 5.25% NaOCl, 2% chlorhexidine, and saline solution (control group) activated by PUI. In all the groups, the lowest maximum depth and percentage values of penetration of the irrigants occurred in the apical root canal third. This result was not only due to the smaller volume and diameter of the dentinal tubules present in the final millimeters of the root canal but also to the greater difficulty the endodontic irrigants had with gaining access to the region, their limited ability to remove the smear layer and the larger quantity of sclerotic dentin present in the area (30).

Mozo, *et al*. (32) evaluated debris removal and the quantity of open dentinal tubules provided by 2.5% NaOCl comparing CI and PUI using Irrisafe ultrasonic inserts ns. 20 and 25, and an n. 25 K-File by scanning electron microscopy images. In the apical root canal third, PUI performed with ultrasonic inserts provided removal of significantly larger quantities of debris and more open dentinal tubules compared with the other methods. Salas, *et al*. (33) evaluated the depth of intratubular penetration of CHX by comparing CI, sonic irrigation (EDDY), and PUI. According to the authors, the best results obtained by PUI could be attributed to some physical factors. Acoustic streaming is a rapid fluid movement in a circular or vortex shape around the vibrating ultrasonic file, which can attract debris and bacteria from the root canal wall. At the same time, cavitation is the formation, behavior, and collapse of bubbles. The destruction of these bubbles close to the root canal walls generates a high-velocity jet directed towards their surfaces, releasing the smear layer and thus enhancing cleaning (12). The high-velocity jet directed towards the root canal walls most likely contributed to the endodontic irrigants’ intratubular penetration. Therefore, in 1988 (34), Cameron reported a rise of the intracanal temperature from 37°C to 45°C close to the instrument’s tip when the irrigant was ultrasonically activated for 30 seconds without replenishment. This time was the same used to perform PUI in the present research. Moreover, the increase in NaOCl temperature during PUI may have helped to reduce its surface tension and may, therefore, have contributed to improving the intratubular penetration of the irrigant in the apical root canal third.

Virdee, *et al*. (17) observed that the intratubular penetration depth values of NaOCl were consistently lower in the apical compared with the middle and cervical root canal thirds, which varied according to the irrigation method (CI, MDA, SI, and PUI), with the concentration of the irrigant (2% and 5.25%) and with the time of exposure (10 and 20 minutes). However, Giardino, *et al*. (16) compared the depth of intratubular penetration of 5.25% NaOCl with and without surfactants and observed no significant statistical differences in the middle and cervical root canal thirds (the apical one was not evaluated). Moreover, Generali, *et al*. (15) found no statistically significant differences concerning the depth values of intratubular penetration of 5.25% NaOCl in the three root canal thirds after MDA and PUI. Variations in the results of the studies are mainly due to the different methodologies used and the alterations that occurred, particularly in the apical dentin from the third decade of life, among which sclerosis and the reduction of tubular density were outstanding (30).

Simultaneous interaction among the three independent variables on the depth values of intratubular penetration of NaOCl was not proved. However, considering the root canal third, the increase in the depth of intratubular penetration values of the irrigant was similar according to the rise in its concentration, irrespective of the irrigation method. Therefore, the volume and diameter of dentinal tubules appeared to have played a more relevant role in the depth of intratubular penetration of NaOCl, than the concentration and irrigation method.

## Conclusions

• The highest depth values of intratubular penetration of NaOCl were observed in the cervical root canal thirds when the product was used at the concentration of 5.25% and in an activated manner (PUI).

• Regarding concentration/irrigation method, concentration/root canal third, and irrigation method/root canal third, the highest penetration depth values of NaOCl were obtained by: 5.25% NaOCl, regardless of the irrigation method (PUI or CI), 5.25% NaOCl in the cervical root canal third, and cervical root canal third, despite of the irrigation method.

• Considering the three independent variables simultaneously, the irrigant’s highest depth values of intratubular penetration were observed in the cervical root canal thirds, using it at the concentration of 5.25%, without statistically significant differences between irrigation methods. In the middle root canal third, there was no statistically significant difference when irrigation methods and concentrations of irrigant were compared. In the apical root canal third, the highest penetration depth values of NaOCl were obtained by PUI, using it at the concentration of 5.25% with significant statistical differences compared to the other combinations. In CI, the highest penetration depth values of the irrigant were also observed using it in the higher concentration, with significant statistical differences compared to the other combinations.

• Analysis of the interaction between the concentration of NaOCl and irrigation method and NaOCl and root canal third on the intratubular penetration depth of the irrigant, indicating their absence. An interaction was identified by considering the irrigation method and root canal third on penetration depth values of the irrigant. Investigating the interaction among all independent variables simultaneously on the depth of intratubular penetration values of NaOCl, no interaction was proven.
